# 4-Methyl-*N*-(4-methyl­benzo­yl)benzene­sulfonamide

**DOI:** 10.1107/S1600536810019756

**Published:** 2010-05-29

**Authors:** P. A. Suchetan, B. Thimme Gowda, Sabine Foro, Hartmut Fuess

**Affiliations:** a Department of Chemistry, Mangalore University, Mangalagangotri 574 199, Mangalore, India; b Institute of Materials Science, Darmstadt University of Technology, Petersenstrasse 23, D-64287 Darmstadt, Germany

## Abstract

In the title compound, C_15_H_15_NO_3_S, the conformation of the N—H bond in the C—SO_2_—NH—C(O) segment is *anti* to the C=O bond. The dihedral angle between the sulfonyl benzene ring and the —SO_2_—NH—C—O segment is 84.9 (1)° and that between the sulfonyl and the benzoyl benzene rings is 89.0 (1)°. In the crystal, inversion dimers linked by pairs of N—H⋯O hydrogen bonds occur.

## Related literature

For background to our study of the effect of ring and side-chain substituents on the crystal structures of *N*-aromatic sulfonamides and for similar structures, see: Gowda *et al.* (2009 ▶); Suchetan *et al.* (2010**a* ▶,*b* ▶,c* ▶).
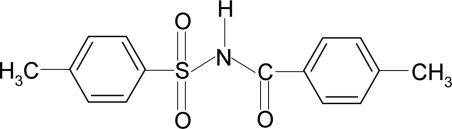


## Experimental

### 

#### Crystal data

C_15_H_15_NO_3_S
*M* *_r_* = 289.34
Monoclinic, 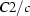

*a* = 23.800 (3) Å
*b* = 5.8518 (7) Å
*c* = 21.027 (3) Å
β = 93.51 (1)°
*V* = 2923.0 (7) Å^3^
*Z* = 8
Mo *K*α radiation
μ = 0.23 mm^−1^
*T* = 299 K
0.34 × 0.24 × 0.12 mm

#### Data collection

Oxford Diffraction Xcalibur diffractometer with a Sapphire CCD detector
Absorption correction: multi-scan (*CrysAlis RED*; Oxford Diffraction, 2009 ▶) *T*_min_ = 0.927, *T*_max_ = 0.973
6121 measured reflections
2994 independent reflections
2162 reflections with *I* > 2σ(*I*)
*R*_int_ = 0.016

#### Refinement

*R*[*F* ^2^ > 2σ(*F* ^2^)] = 0.043
*wR*(*F* ^2^) = 0.118
*S* = 1.04
2994 reflections
186 parameters
1 restraint
H atoms treated by a mixture of independent and constrained refinement
Δρ_max_ = 0.22 e Å^−3^
Δρ_min_ = −0.25 e Å^−3^

### 

Data collection: *CrysAlis CCD* (Oxford Diffraction, 2009 ▶); cell refinement: *CrysAlis RED* (Oxford Diffraction, 2009 ▶); data reduction: *CrysAlis RED*; program(s) used to solve structure: *SHELXS97* (Sheldrick, 2008 ▶); program(s) used to refine structure: *SHELXL97* (Sheldrick, 2008 ▶); molecular graphics: *PLATON* (Spek, 2009 ▶); software used to prepare material for publication: *SHELXL97*.

## Supplementary Material

Crystal structure: contains datablocks I, global. DOI: 10.1107/S1600536810019756/bq2214sup1.cif

Structure factors: contains datablocks I. DOI: 10.1107/S1600536810019756/bq2214Isup2.hkl

Additional supplementary materials:  crystallographic information; 3D view; checkCIF report

## Figures and Tables

**Table 1:** Hydrogen-bond geometry (Å, °)

*D*—H⋯*A*	*D*—H	H⋯*A*	*D*⋯*A*	*D*—H⋯*A*
N1—H1*N*⋯O1^i^	0.85 (1)	2.19 (1)	3.013 (2)	165 (2)

Symmetry code: (i) 
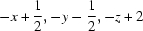
.

**Fig. 1.**
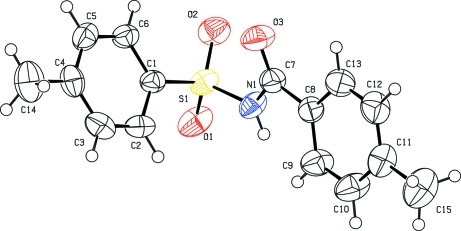
Molecular structure of the title compound, showing the atom-labeling scheme. Displacement ellipsoids are drawn at the 50% probability level.

**Fig. 2.**
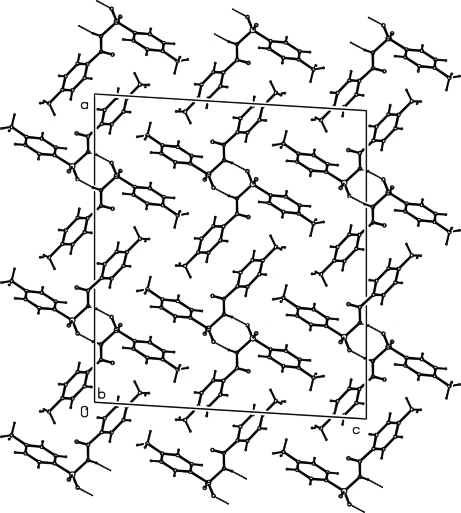
Molecular packing in the title compound. Hydrogen bonds are shown as dashed lines.

## supplementary crystallographic information

### Comment

As a part of studying the effect of ring and the side chain substituents on the crystal structures of *N*-aromatic sulfonamides (Gowda *et al.*, 2009; Suchetan *et al.*, 2010*a,b,c*), the structure of 4-Methyl-*N*-(4-methylbenzoyl)-benzenesulfonamide has been determined. The conformation of the N—H bond in the C—SO_2_—NH—C(O) segment is *anti* to the C=O bond (Fig.1), similar to those observed in *N*-(benzoyl)benzenesulfonamide (II) (Gowda *et al.*, 2009), *N*-(4-methylbenzoyl)-4-chlorobenzenesulfonamide (III) (Suchetan *et al.*, 2010*c*), *N*-(4-chlorobenzoyl)-4- methylbenzenesulfonamide (IV) (Suchetan *et al.*, 2010*a*), and *N*-(4-chlorobenzoyl)-4-chlorobenzenesulfonamide (V) (Suchetan *et al.*, 2010*b*).

The molecules are twisted at the *S* atoms with the torsional angle of 62.0 (2)°, compared to the values of -66.9 (3)° in (II), 69.0 (2)° in (III), 67.1 (2)° (molecule 1) & 67.7 (2)° (molecule 2) in (IV) and 67.5 (3)° in (V). The dihedral angle between the sulfonyl benzene ring and the —SO_2_—NH—C—O segment is 84.9 (1)°, compared to the values of 86.5 (1)° in (II), 77.2 (1)° in (III), 83.6 (1)° (molecule 1) and 81.0 (1)° (molecule 2) in (IV) and 79.0 (1)° in (V) The dihedral angle between the sulfonyl and the benzoyl benzene rings is 89.0 (1)°, compared to the values of 80.3 (1)° in (II), 89.5 (1)° in (III), 81.0 (1)° (molecule 1) and 76.3 (1)° (molecule 2) in (IV) and 85.6 (1)° in (V). The packing of molecules in the crystal linked by N—H···O hydrogen bonds (Table 1) is shown in Fig. 2.

### Experimental

The title compound was prepared by refluxing a mixture of 4-methylbenzoic acid, 4-methylbenzenesulfonamide and phosphorous oxy chloride for 3 h on a water bath. The resultant mixture was cooled and poured into ice cold water. The solid obtained was filtered, washed thoroughly with water and then dissolved in sodium bicarbonate solution. The compound was later reprecipitated by acidifying the filtered solution with dilute HCl. It was filtered, dried and recrystallized. Rod like colorless single crystals of the title compound used in x-ray diffraction studies were obtained by slow evaporation of its toluene solution at room temperature.

### Refinement

The H atom of the NH group was located in a difference map and later restrained to N—H = 0.86 (1)%A. The other H atoms were positioned with idealized geometry using a riding model with C—H = 0.93–0.96Å. All H atoms were refined with isotropic displacement parameters (set to 1.2 times of the *U*_eq_ of the parent atom).

### Crystal data

**Table:**

C_15_H_15_NO_3_S	*F*(000) = 1216
*M**_r_* = 289.34	*D*_x_ = 1.315 Mg m^−^^3^
Monoclinic, *C*2/*c*	Mo *K*α radiation, λ = 0.71073 Å
Hall symbol: -C 2yc	Cell parameters from 2019 reflections
*a* = 23.800 (3) Å	θ = 2.5–27.8°
*b* = 5.8518 (7) Å	µ = 0.23 mm^−^^1^
*c* = 21.027 (3) Å	*T* = 299 K
β = 93.51 (1)°	Rod, colourless
*V* = 2923.0 (7) Å^3^	0.34 × 0.24 × 0.12 mm
*Z* = 8	

### Data collection

**Table:**

Oxford Diffraction Xcalibur diffractometer with a Sapphire CCD detector	2994 independent reflections
Radiation source: fine-focus sealed tube	2162 reflections with *I* > 2σ(*I*)
graphite	*R*_int_ = 0.016
Rotation method data acquisition using ω and phi scans	θ_max_ = 26.4°, θ_min_ = 2.5°
Absorption correction: multi-scan (*CrysAlis RED*; Oxford Diffraction, 2009)	*h* = −16→29
*T*_min_ = 0.927, *T*_max_ = 0.973	*k* = −7→5
6121 measured reflections	*l* = −26→26

### Refinement

**Table:**

Refinement on *F*^2^	Primary atom site location: structure-invariant direct methods
Least-squares matrix: full	Secondary atom site location: difference Fourier map
*R*[*F*^2^ > 2σ(*F*^2^)] = 0.043	Hydrogen site location: inferred from neighbouring sites
*wR*(*F*^2^) = 0.118	H atoms treated by a mixture of independent and constrained refinement
*S* = 1.04	*w* = 1/[σ^2^(*F*_o_^2^) + (0.0532*P*)^2^ + 1.827*P*] where *P* = (*F*_o_^2^ + 2*F*_c_^2^)/3
2994 reflections	(Δ/σ)_max_ = 0.001
186 parameters	Δρ_max_ = 0.22 e Å^−^^3^
1 restraint	Δρ_min_ = −0.25 e Å^−^^3^

### Special details

**Table:**

Experimental. CrysAlis RED (Oxford Diffraction, 2009) Empirical absorption correction using spherical harmonics, implemented in SCALE3 ABSPACK scaling algorithm.
Geometry. All e.s.d.'s (except the e.s.d. in the dihedral angle between two l.s. planes) are estimated using the full covariance matrix. The cell e.s.d.'s are taken into account individually in the estimation of e.s.d.'s in distances, angles and torsion angles; correlations between e.s.d.'s in cell parameters are only used when they are defined by crystal symmetry. An approximate (isotropic) treatment of cell e.s.d.'s is used for estimating e.s.d.'s involving l.s. planes.
Refinement. Refinement of *F*^2^ against ALL reflections. The weighted *R*-factor *wR* and goodness of fit *S* are based on *F*^2^, conventional *R*-factors *R* are based on *F*, with *F* set to zero for negative *F*^2^. The threshold expression of *F*^2^ > σ(*F*^2^) is used only for calculating *R*-factors(gt) *etc*. and is not relevant to the choice of reflections for refinement. *R*-factors based on *F*^2^ are statistically about twice as large as those based on *F*, and *R*- factors based on ALL data will be even larger.

### Fractional atomic coordinates and isotropic or equivalent isotropic displacement parameters (Å^2^)

**Table:**

	*x*	*y*	*z*	*U*_iso_*/*U*_eq_	
C1	0.29392 (8)	−0.0818 (3)	0.85224 (9)	0.0411 (5)	
C2	0.31864 (9)	−0.2944 (3)	0.85702 (11)	0.0531 (6)	
H2	0.3174	−0.3791	0.8943	0.064*	
C3	0.34511 (10)	−0.3795 (4)	0.80590 (12)	0.0585 (6)	
H3	0.3622	−0.5222	0.8092	0.070*	
C4	0.34694 (9)	−0.2578 (4)	0.74944 (11)	0.0541 (6)	
C5	0.32105 (9)	−0.0467 (4)	0.74553 (10)	0.0517 (5)	
H5	0.3214	0.0362	0.7078	0.062*	
C6	0.29473 (8)	0.0436 (4)	0.79654 (9)	0.0450 (5)	
H6	0.2778	0.1867	0.7935	0.054*	
C7	0.36098 (9)	0.1360 (3)	0.97561 (10)	0.0471 (5)	
C8	0.40479 (9)	0.0804 (4)	1.02664 (10)	0.0462 (5)	
C9	0.40501 (10)	−0.1186 (4)	1.06220 (12)	0.0611 (6)	
H9	0.3757	−0.2231	1.0560	0.073*	
C10	0.44864 (10)	−0.1621 (5)	1.10682 (12)	0.0689 (7)	
H10	0.4482	−0.2970	1.1302	0.083*	
C11	0.49272 (9)	−0.0126 (5)	1.11791 (11)	0.0596 (6)	
C12	0.49263 (10)	0.1834 (5)	1.08157 (12)	0.0646 (7)	
H12	0.5221	0.2868	1.0877	0.077*	
C13	0.44978 (10)	0.2293 (4)	1.03648 (11)	0.0592 (6)	
H13	0.4510	0.3619	1.0123	0.071*	
C14	0.37698 (12)	−0.3559 (6)	0.69460 (13)	0.0850 (9)	
H14A	0.3637	−0.5083	0.6859	0.102*	
H14B	0.4167	−0.3594	0.7054	0.102*	
H14C	0.3696	−0.2624	0.6575	0.102*	
C15	0.53956 (11)	−0.0630 (6)	1.16734 (14)	0.0872 (9)	
H15A	0.5749	−0.0641	1.1476	0.105*	
H15B	0.5333	−0.2096	1.1861	0.105*	
H15C	0.5403	0.0525	1.1998	0.105*	
N1	0.31089 (7)	0.0168 (3)	0.97639 (8)	0.0500 (5)	
H1N	0.3035 (9)	−0.074 (3)	1.0061 (8)	0.060*	
O1	0.21990 (6)	−0.1306 (3)	0.93756 (8)	0.0651 (5)	
O2	0.24436 (7)	0.2579 (3)	0.90537 (8)	0.0620 (4)	
O3	0.36862 (7)	0.2731 (3)	0.93398 (8)	0.0666 (5)	
S1	0.26113 (2)	0.02819 (10)	0.91814 (2)	0.04836 (18)	

### Atomic displacement parameters (Å^2^)

**Table:**

	*U*^11^	*U*^22^	*U*^33^	*U*^12^	*U*^13^	*U*^23^
C1	0.0392 (10)	0.0389 (11)	0.0450 (11)	−0.0041 (8)	−0.0001 (8)	0.0054 (8)
C2	0.0582 (13)	0.0417 (12)	0.0592 (13)	−0.0001 (10)	0.0032 (11)	0.0135 (10)
C3	0.0579 (14)	0.0411 (12)	0.0763 (17)	0.0022 (11)	0.0026 (12)	−0.0039 (11)
C4	0.0445 (12)	0.0611 (14)	0.0562 (13)	−0.0105 (11)	0.0002 (10)	−0.0154 (11)
C5	0.0497 (12)	0.0622 (14)	0.0424 (11)	−0.0088 (11)	−0.0032 (9)	0.0050 (10)
C6	0.0425 (11)	0.0435 (11)	0.0483 (11)	−0.0014 (9)	−0.0034 (9)	0.0080 (9)
C7	0.0521 (12)	0.0442 (11)	0.0453 (11)	−0.0004 (10)	0.0062 (9)	0.0027 (10)
C8	0.0467 (11)	0.0484 (12)	0.0441 (11)	−0.0049 (10)	0.0075 (9)	0.0000 (9)
C9	0.0481 (13)	0.0610 (14)	0.0729 (16)	−0.0143 (11)	−0.0058 (11)	0.0174 (12)
C10	0.0548 (14)	0.0765 (17)	0.0741 (17)	−0.0099 (13)	−0.0050 (12)	0.0262 (14)
C11	0.0418 (12)	0.0820 (18)	0.0550 (13)	−0.0042 (12)	0.0026 (10)	−0.0007 (12)
C12	0.0518 (13)	0.0727 (17)	0.0690 (16)	−0.0226 (12)	0.0019 (12)	−0.0052 (13)
C13	0.0609 (14)	0.0560 (14)	0.0608 (14)	−0.0156 (12)	0.0066 (12)	0.0044 (11)
C14	0.0774 (19)	0.102 (2)	0.0768 (19)	0.0039 (17)	0.0133 (15)	−0.0287 (17)
C15	0.0535 (15)	0.127 (3)	0.0792 (19)	−0.0048 (17)	−0.0084 (14)	0.0070 (18)
N1	0.0467 (10)	0.0637 (12)	0.0400 (9)	−0.0048 (9)	0.0045 (8)	0.0117 (8)
O1	0.0416 (8)	0.0903 (12)	0.0635 (10)	−0.0085 (8)	0.0047 (7)	0.0256 (9)
O2	0.0631 (10)	0.0607 (10)	0.0629 (10)	0.0204 (8)	0.0098 (8)	0.0071 (8)
O3	0.0687 (11)	0.0632 (10)	0.0674 (10)	−0.0093 (8)	0.0011 (8)	0.0246 (9)
S1	0.0402 (3)	0.0579 (4)	0.0472 (3)	0.0026 (3)	0.0052 (2)	0.0111 (3)

### Geometric parameters (Å, °)

**Table:**

C1—C2	1.377 (3)	C9—H9	0.9300
C1—C6	1.383 (3)	C10—C11	1.375 (3)
C1—S1	1.754 (2)	C10—H10	0.9300
C2—C3	1.372 (3)	C11—C12	1.378 (3)
C2—H2	0.9300	C11—C15	1.506 (3)
C3—C4	1.387 (3)	C12—C13	1.376 (3)
C3—H3	0.9300	C12—H12	0.9300
C4—C5	1.381 (3)	C13—H13	0.9300
C4—C14	1.507 (3)	C14—H14A	0.9600
C5—C6	1.380 (3)	C14—H14B	0.9600
C5—H5	0.9300	C14—H14C	0.9600
C6—H6	0.9300	C15—H15A	0.9600
C7—O3	1.209 (2)	C15—H15B	0.9600
C7—N1	1.382 (3)	C15—H15C	0.9600
C7—C8	1.486 (3)	N1—S1	1.6521 (18)
C8—C9	1.384 (3)	N1—H1N	0.847 (10)
C8—C13	1.386 (3)	O1—S1	1.4293 (15)
C9—C10	1.380 (3)	O2—S1	1.4231 (16)
			
C2—C1—C6	120.9 (2)	C10—C11—C12	117.4 (2)
C2—C1—S1	118.86 (16)	C10—C11—C15	121.2 (2)
C6—C1—S1	120.28 (16)	C12—C11—C15	121.4 (2)
C3—C2—C1	119.0 (2)	C13—C12—C11	121.4 (2)
C3—C2—H2	120.5	C13—C12—H12	119.3
C1—C2—H2	120.5	C11—C12—H12	119.3
C2—C3—C4	121.6 (2)	C12—C13—C8	120.9 (2)
C2—C3—H3	119.2	C12—C13—H13	119.5
C4—C3—H3	119.2	C8—C13—H13	119.5
C5—C4—C3	118.2 (2)	C4—C14—H14A	109.5
C5—C4—C14	121.7 (2)	C4—C14—H14B	109.5
C3—C4—C14	120.0 (2)	H14A—C14—H14B	109.5
C6—C5—C4	121.2 (2)	C4—C14—H14C	109.5
C6—C5—H5	119.4	H14A—C14—H14C	109.5
C4—C5—H5	119.4	H14B—C14—H14C	109.5
C5—C6—C1	119.0 (2)	C11—C15—H15A	109.5
C5—C6—H6	120.5	C11—C15—H15B	109.5
C1—C6—H6	120.5	H15A—C15—H15B	109.5
O3—C7—N1	120.8 (2)	C11—C15—H15C	109.5
O3—C7—C8	122.6 (2)	H15A—C15—H15C	109.5
N1—C7—C8	116.65 (18)	H15B—C15—H15C	109.5
C9—C8—C13	118.1 (2)	C7—N1—S1	123.40 (14)
C9—C8—C7	123.63 (19)	C7—N1—H1N	122.9 (16)
C13—C8—C7	118.15 (19)	S1—N1—H1N	113.7 (16)
C10—C9—C8	120.0 (2)	O2—S1—O1	118.59 (10)
C10—C9—H9	120.0	O2—S1—N1	110.99 (10)
C8—C9—H9	120.0	O1—S1—N1	103.62 (9)
C11—C10—C9	122.2 (2)	O2—S1—C1	109.32 (9)
C11—C10—H10	118.9	O1—S1—C1	109.49 (10)
C9—C10—H10	118.9	N1—S1—C1	103.73 (9)
			
C6—C1—C2—C3	1.1 (3)	C9—C10—C11—C15	179.4 (3)
S1—C1—C2—C3	−178.98 (17)	C10—C11—C12—C13	0.8 (4)
C1—C2—C3—C4	−0.8 (3)	C15—C11—C12—C13	−180.0 (2)
C2—C3—C4—C5	−0.3 (3)	C11—C12—C13—C8	0.8 (4)
C2—C3—C4—C14	179.3 (2)	C9—C8—C13—C12	−1.8 (3)
C3—C4—C5—C6	1.0 (3)	C7—C8—C13—C12	−177.8 (2)
C14—C4—C5—C6	−178.6 (2)	O3—C7—N1—S1	6.9 (3)
C4—C5—C6—C1	−0.7 (3)	C8—C7—N1—S1	−170.96 (15)
C2—C1—C6—C5	−0.4 (3)	C7—N1—S1—O2	−55.3 (2)
S1—C1—C6—C5	179.71 (15)	C7—N1—S1—O1	176.38 (18)
O3—C7—C8—C9	−159.1 (2)	C7—N1—S1—C1	62.01 (19)
N1—C7—C8—C9	18.7 (3)	C2—C1—S1—O2	173.39 (16)
O3—C7—C8—C13	16.7 (3)	C6—C1—S1—O2	−6.7 (2)
N1—C7—C8—C13	−165.5 (2)	C2—C1—S1—O1	−55.16 (18)
C13—C8—C9—C10	1.3 (4)	C6—C1—S1—O1	124.75 (17)
C7—C8—C9—C10	177.1 (2)	C2—C1—S1—N1	54.94 (18)
C8—C9—C10—C11	0.3 (4)	C6—C1—S1—N1	−125.15 (17)
C9—C10—C11—C12	−1.3 (4)		

### Hydrogen-bond geometry (Å, °)

**Table:**

*D*—H···*A*	*D*—H	H···*A*	*D*···*A*	*D*—H···*A*
N1—H1N···O1^i^	0.85 (1)	2.19 (1)	3.013 (2)	165 (2)

Symmetry codes: (i) −*x*+1/2, −*y*−1/2, −*z*+2.
